# Two Distinct Scene-Processing Networks Connecting Vision and Memory

**DOI:** 10.1523/ENEURO.0178-16.2016

**Published:** 2016-10-24

**Authors:** Christopher Baldassano, Andre Esteva, Li Fei-Fei, Diane M. Beck

**Affiliations:** 1 Department of Computer Science, Stanford University, Stanford, California 94305; 2 Department of Electrical Engineering, Stanford University, Stanford, California 94305; 3 Department of Psychology, University of Illinois at Urbana-Champaign, Champaign, Illinois 61820; 4 Beckman Institute, University of Illinois at Urbana-Champaign, Champaign, Illinois 61820

**Keywords:** memory, networks, scene, vision

## Abstract

A number of regions in the human brain are known to be involved in processing natural scenes, but the field has lacked a unifying framework for understanding how these different regions are organized and interact. We provide evidence from functional connectivity and meta-analyses for a new organizational principle, in which scene processing relies upon two distinct networks that split the classically defined parahippocampal place area (PPA). The first network of strongly connected regions consists of the occipital place area/transverse occipital sulcus and posterior PPA, which contain retinotopic maps and are not strongly coupled to the hippocampus at rest. The second network consists of the caudal inferior parietal lobule, retrosplenial complex, and anterior PPA, which connect to the hippocampus (especially anterior hippocampus), and are implicated in both visual and nonvisual tasks, including episodic memory and navigation. We propose that these two distinct networks capture the primary functional division among scene-processing regions, between those that process visual features from the current view of a scene and those that connect information from a current scene view with a much broader temporal and spatial context. This new framework for understanding the neural substrates of scene-processing bridges results from many lines of research, and makes specific functional predictions.

## Visual Abstract

**Figure:**
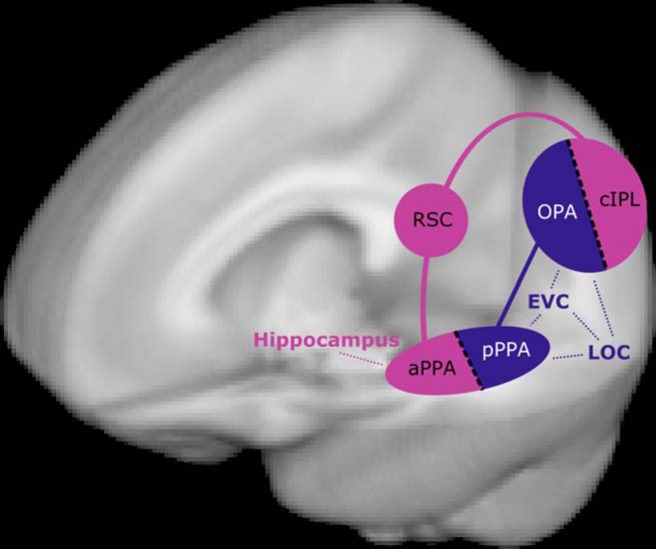

## Significance Statement

There are a number of brain regions that only show high levels of activity for full photographic scenes, not individual objects. By examining their relationships to each other and the rest of the brain, we argue that there are two types of scene-processing regions that belong to two separate networks. One network, which overlaps most of the visual system, processes visual features of the current view of the world, such as spatial layout. Another network, which is connected to long-term memory, puts this moment-by-moment information in context, allowing us to navigate through environments and remember past events in familiar locations. These two groups of brain regions cooperate to help us understand the world and our place in it.

## Introduction

Natural scene perception has been shown to rely upon a distributed set of cortical regions, including the parahippocampal place area (PPA; Epstein and Kanwisher, 1998), retrosplenial complex (RSC; O’Craven and Kanwisher, 2000), and the occipital place area [OPA; also called the transverse occipital sulcus (TOS); Nakamura et al., 2000; Hasson et al., 2003]. More recent work has suggested that the picture is even more complicated, with multiple subdivisions within PPA and the possible involvement of the parietal lobe (Baldassano et al., 2013). Although there has been substantial progress in understanding the functional properties of each of these regions and the differences between them, the field has lacked a coherent framework for summarizing the overall architecture of the human scene-processing system.

There is a long history of proposals for partitioning the visual system into separable components with different functions, such as spatial frequency channels (Campbell and Robson, 1968); what versus where/how pathways (Mishkin et al., 1983; Kravitz et al., 2011); or magnocellular, parvocellular, and koniocellular streams (Kaplan, 2004). With respect to natural scene perception, one can imagine at least two separable functions: processing the specific visual features present in the current glance of a scene, and connecting that to the stable, high-level knowledge of where the place exists in the world, what has happened here in the past, and what possible actions we could take here in the future. For most cognitive and physical tasks we undertake in real-world places, the specific visual attributes we perceive are just a means to this end, of recalling and updating information about the physical environment; “the essential feature of a landmark is not its design, but the place it holds in a city's memory” (Muschamp, 2006). The connection between place and memory has been recognized for thousands of years, reflected in the ancient Greek “method of loci” that strengthens a memory sequence by associating it with physical locations (Yates, 1966).

To determine whether moment-by-moment visual processing versus dependence on past experience is a major organizing principle of the brain, we take a data-driven approach to identifying scene-sensitive regions and clustering cortical connectivity. We first aggregate local high-resolution resting-state connectivity information into spatially coherent parcels, in order to increase signal to noise and obtain more interpretable units than individual voxels. We then apply hierarchical clustering to show that there exists a natural division in posterior human cortex that splits scene-related regions into two separate, bilaterally symmetric networks. The posterior network includes OPA and the posterior portion of PPA (retinotopic maps PHC1 and PHC2), while the anterior network is composed of the RSC, anterior PPA (aPPA), and the caudal inferior parietal lobule (cIPL). We then show that these two networks differ in their connectivity to the hippocampus, with the anterior network exhibiting much higher resting-state hippocampal coupling (especially to anterior hippocampus), suggesting that memory- and navigation-related functions are primarily restricted to the anterior network. We provide supporting evidence for this functional division from a reverse-inference meta-analysis of previous results from visual, memory, and navigation studies, and an atlas of retinotopic maps.

Based on these results, as well as a review of previous work, we propose that scene processing is fundamentally divided into two collaborating but distinct networks, with one focused on the visual features of a scene image and the other related to contextual retrieval and navigation. Under this framework, scene perception is less the function of a unified set of distributed neural machinery and more of “an ongoing dialogue between the material and symbolic aspects of the past and the continuously unfolding present” (Baker, 2012).

## Materials and Methods

### Imaging data

The majority of the data used in this study were obtained from the Human Connectome Project (HCP), which provides detailed documentation on the experimental and acquisition parameters for these datasets (Van Essen et al., 2013). We provide an overview of these datasets below.

**Figure 1.**
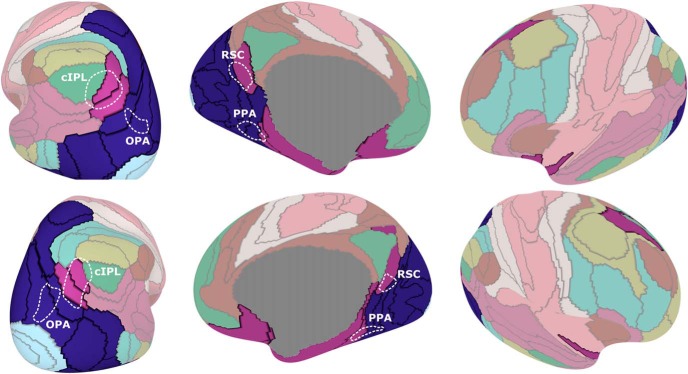
Connectivity clustering of cortical parcels. The cortex was first grouped into 172 local parcels (black lines), such that the surface vertices in each parcel had similar connectivity properties. Performing a second-level hierarchical clustering on these parcels identified distributed networks of strongly connected parcels (parcel colors denote their network membership). Scene-related regions of interest (identified using standard scene localizers in a separate group of subjects) are split across two networks, which are largely symmetric across left (top row) and right (bottom row) hemispheres. OPA and posterior PPA overlap with a posterior network (dark blue) that covers all of visual cortex outside the foveal confluence, while cIPL, RSC, and aPPA overlap with an anterior network (magenta) that covers much of the default mode network.

**Figure 2.**
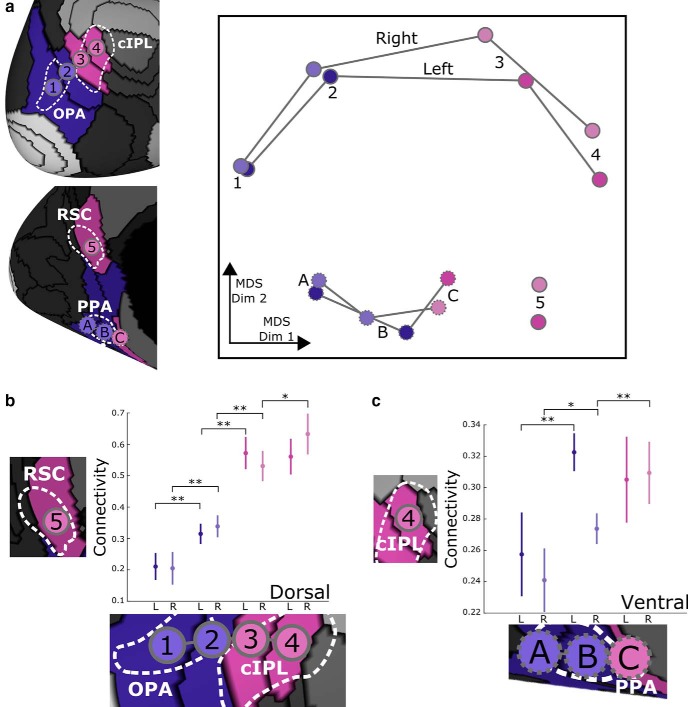
Connectivity shifts across the network border. ***a***, Using classic multidimensional scaling (MDS), we can visualize the connectivity structure among the eight parcels overlapping with scene-related regions (darker/lighter shading denotes left/right hemisphere). The first MDS dimension shows a parallel transition along both dorsal and ventral paths from parcels overlapping OPA and pPPA to those overlapping cIPL, RSC, and aPPA. ***b***, Connectivity between dorsal parcels and the medial RSC parcel increases markedly near the OPA/cIPL border. ***b***, Ventral parcels also show a shift in network connectivity properties, with increasing connectivity to the most anterior cIPL parcel as we move from pPPA to aPPA. Error bars are 95% confidence intervals across subjects, *p < 0.05, **p < 0.01.

**Figure 3.**
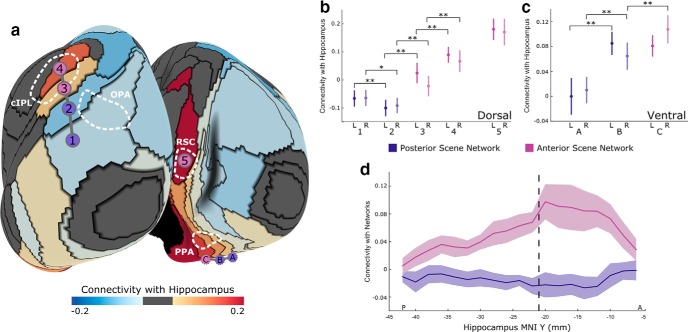
Connectivity between network parcels and the hippocampus. ***a***, For each parcel in the anterior and posterior scene networks, we computed its resting-state connectivity with the hippocampus, showing a striking increase in hippocampal activity for anterior network parcels overlapping with cIPL, RSC, and aPPA (magenta circles) compared with posterior network parcels (blue circles). ***b***, Along the dorsal network boundary, hippocampal activity first dips slightly and then increases substantially, becoming strongest in the most anterior parcel intersecting cIPL (and is also high in RSC). ***c***, Ventrally along parcels overlapping with PPA, we observe a similar increasing posterior-to-anterior gradient in connectivity. ***d***, Computing the connectivity between each coronal slice of the hippocampus and the two scene networks shows that this increased coupling to the anterior network is present throughout the hippocampus, but is especially pronounced in anterior hippocampus (MNI coordinate *y* > −21 mm). Error bars are 95% confidence intervals across subjects. *p < 0.05, **p < 0.01.

The group-level functional connectivity data were derived from the 468-subject group–principal component analysis (PCA) eigenmaps, distributed with the June 2014 “500 Subjects” HCP data release. Resting-state fMRI data were acquired over four sessions (14 min, 33 s each), while subjects fixated on a bright cross-hair on a dark background, using a multiband sequence to achieve a TR of 720 ms at 2.0 mm isotropic resolution (59,412 surface vertices). These time courses were cleaned using the Oxford Centre for Functional MRI of the Brain independent component analysis-based Xnoiseifier (FIX; Salimi-Khorshidi et al., 2014), and then the top 4500 eigenvectors for each vertex were estimated across all subjects using Group–PCA (Smith et al., 2014). These data were used to perform the parcellation and network clustering, and to generate whole-brain maps (Figs. 1, 2a, 3*a* )

Because using the full dataset in its entirety would be computationally challenging to assess statistically, we performed more detailed analyses on a subset of 20 subjects (Figs. 2b,*c*, 3*b–d* ). For 20 subjects within the “500 Subjects” release with complete data (subject identifications 101006, 101107, 101309, 102008, 102311, 103111, 104820, 105014, 106521, 107321, 107422, 108121, 108323, 108525, 108828, 109123, 109325, 111413, 113922, and 120515), we created individual subject resting-state datasets by concatenating their four resting-state sessions (after removing the per-run means).

We identified group-level scene localizers (used only as functional landmarks) from a separate set of 24 subjects (see below). Subjects viewed blocks of stimuli from up to six categories: child faces, adult faces, indoor scenes, outdoor scenes, objects (abstract sculptures with no semantic meaning), and scrambled objects. Functional data were acquired on one of two GE MR 750 3 T scanners, with an in-place resolution of 1.56 mm, a slice thickness of 3 mm (with 1 mm gap), and a TR of 2 s; a high-resolution (1 mm isotropic) spoiled gradient-recalled acquisition in a steady state structural scan was also acquired to allow for transformation to MNI space.

The cIPL was defined using the Eickhoff–Zilles PGp probabilistic cytoarchitectonic map (Eickhoff et al., 2005; Baldassano et al., 2013). The hippocampus was divided into anterior and posterior subregions at MNI coordinate *y* = −21, consistent with previous studies (Poppenk et al., 2013; Zeidman et al. 2015).

### Subjects

Scene-localizer data were collected from 24 subjects (6 females; age range, 22–32, including one of the authors). Subjects were in good health with no history of psychiatric or neurological diseases, and with normal or corrected-to-normal vision. The experimental protocol was approved by the institutional review board of Stanford University. Subjects were recruited only at Stanford University and gave their written informed consent.

### Resting-state parcellation

The 468-subject eigenmaps distributed by the HCP are approximately equal to performing a singular value decomposition on the concatenated time courses of all 468 subjects, and then retaining the right singular values scaled by their eigenvalues (Smith et al., 2014). This allows us to treat these eigenmaps as pseudo-time courses, since dot products (and thus Pearson correlations) between eigenmaps approximate the dot products between the original voxel time courses. We generated a voxel-level functional connectivity matrix by correlating the group-level eigenmaps for every pair of voxels and applying the Fisher *z*-transform (hyperbolic arctangent). We parcellated this 59,412 × 59,412 matrix into contiguous regions, using a generative probabilistic model (Baldassano et al., 2015). This method finds a parcellation of the cortex such that the connectivity properties within each parcel are as uniform as possible, making multiple passes over the dataset to fine-tune the parcel borders. We set the scaling hyperparameter $\sigma_{0}^{2}=3000$ to produce a manageable number of parcels, but our clustering results are similar for a wide range of settings for $\sigma_{0}^{2}$ (producing between 140 and 216 parcels).

### Scene localizers

To identify PPA, RSC, and OPA, we deconvolved the localizer data from the 24 localizer subjects using the standard block hemodynamic model in AFNI (Cox, 1996), with faces, scenes, objects, and scrambled objects as regressors. The scenes > objects *t* statistic was used to define PPA (top 300 voxels near the parahippocampal gyrus), RSC (top 200 voxels near retrosplenial cortex), and OPA (top 200 voxels near the transverse occipital sulcus), with mask sizes chosen conservatively based on typical ROI volumes (Golarai et al., 2007). The ROI masks were then transformed to MNI space, summed across all subjects, and mapped to the closest vertices on the group cortical surface. The group-level ROI was then manually annotated as the cluster of highest overlap between the subject ROI masks. These ROIs are consistent with typical definitions in the literature (Julian et al., 2012).

### Parcel-to-parcel and hippocampal functional connectivity

Given a parcellation, we computed the group-level connectivity between a pair of regions by taking the mean over all eigenmaps in each region, then correlating these mean eigenmaps (which, as described above, can be treated as pseudo-time courses) and applying the Fisher *z*-transform (hyperbolic arctangent). We computed subject-level connectivity in the same way, using the resting-state time course for each voxel rather than the eigenmap.

Connectivity between cortical parcels and the hippocampus was computed similarly, using eigenmaps (for group data) or time courses (for subject data) extracted from the hippocampal volume data distributed by the HCP. In order to focus on hippocampal connectivity differences among parcels, we used the mean gray time course regression version of the group data and regressed out the global time course from the subject data.

### Network clustering

The 172 × 172 parcel functional connectivity matrix was converted into a distance matrix by subtracting every entry from the maximum entry. Hierarchical ward clustering (unconstrained by parcel position) was applied to the distance matrix to compute a hard clustering into 10 networks. After identifying the 16 parcels (8 per hemisphere) overlapping with scene-related regions, we computed a similar distance matrix for these parcels (subtracting every entry from the maximum entry) and applied classical multidimensional scaling to yield a two-dimensional visualization of its structure.

### Meta-analysis and retinotopic field maps

Two reverse-inference meta-analyses were performed using the NeuroSynth website (Yarkoni et al., 2011). NeuroSynth is a set of open-source python tools for automatically extracting data from fMRI studies and computing activation likelihood maps, and the website hosts these tools (and associated datasets) for public use. Supplying a key word query identifies all studies whose abstract contains that key word, and then analyzes the activations reported in these queried studies. In addition to standard “forward inference” maps giving the probability *p*(activation|query) that a voxel will be activated in these studies, NeuroSynth generates “reverse-inference” maps giving the probability *p*(query|activation) that a voxel activation came specifically from this query set. Voxels appearing in the reverse-inference map, therefore, appear more often in the query set relative to the full set of (>10,000) fMRI studies in the database. This accounts for base rate differences in how often activation is observed in different brain regions.

Our meta-analyses can be viewed on-line at http://neurosynth.org/analyses/custom/dda0e003-efd0-4cfa/ and http://neurosynth.org/analyses/custom/9e6df59d-02df-4357/. The first used the query “scene,” and consisted of 47 studies. Manual inspection of all studies confirmed that they all studied the perception of environments, and 45 of 47 studies involved the presentation of visual scenes. The second meta-analysis used the query “episodic memory OR navigation OR past future,” which returned 125 studies that were nonoverlapping with the first query.

A volumetric group-level probabilistic atlas (Wang et al., 2014) was used to define retinotopic field maps. We computed the total probability mass of each map that fell within one of our two networks or in other regions of the cortex, and then normalized the sum of the three values to 100%. For visualization, the probability that a voxel belongs to any field map was computed as $1{-}_{i}^{\Pi }(1-p_{i})$, where *p_i_* is the probability that the voxel falls within field map *i*.

## Results

Our primary dataset is a 1.8 billion element resting-state connectivity matrix distributed by the Human Connectome Project (Van Essen et al., 2013), which estimates the time course correlation between every pair of locations in the brain at 2 mm resolution based on a group of 468 subjects. Since we wish to understand the large-scale structure of visual cortex, it is helpful to abstract away from individual voxels and study the functional and connectivity properties of larger parcels. Rather than imposing a parcellation based on specific regions of interest, we used a data-driven approach to produce spatially coherent parcels tiling the cortical surface in a way that retains as much information as possible from the full connectivity matrix. This parcellation consists of 172 regions across both hemispheres, each of which contains surface vertices that all have very similar connectivity patterns with the rest of the brain. The connectivity matrix among these 172 parcels captures >76% of the variance in the original connectivity matrix, despite being dramatically smaller (by five orders of magnitude).

### Clustering parcels into networks

To determine how these local parcels are organized into distributed networks, we performed hierarchical clustering to group together parcels with high functional connectivity (regardless of their spatial position). These networks are remarkably similar between hemispheres (despite not being constrained to be symmetric), as shown in the 10-network clustering in Figure 1.

Which of these networks are directly related to scene perception? We used data from a standard localizer in a separate group of subjects to define group-level regions of interest for scene-selective regions OPA, PPA, and RSC. We also anatomically identified cIPL as was done in a previous study (Baldassano et al., 2013), since this region has been shown to have functional connections to scene regions.

We found that these scene ROIs fell almost entirely onto two of the connectivity networks. A posterior network (dark blue), overlapping OPA and posterior PPA (pPPA), covered all of visual cortex outside of an early foveal cluster. An anterior network (magenta), overlapping cIPL, RSC, and anterior PPA, covered a parietal/medial-temporal network that includes anterior temporal and orbitofrontal parcels. This corresponds to a portion of known default mode regions, with other default mode regions being grouped into a separate network (green); a similar fractionation of the default mode has been proposed previously (Andrews-Hanna et al., 2010). Within the PPA, this anterior/posterior split occurred at approximately MNI coordinate *y* = −42 mm, with both segments of the PPA falling largely in the collateral sulcus and extending onto the parahippocampal gyrus.

We can visualize the connectivity differences among the parcels overlapping with scene-related regions using classic multidimensional scaling (Fig. 2a), which shows that the network clustering captures the primary dimension of variance in connectivity properties, separating the most posterior parcels overlapping OPA and pPPA from the most anterior parcels overlapping cIPL, RSC, and aPPA. To evaluate the reliability of this shift in connectivity properties within individual subjects, we measured the functional connectivity between these parcels and a reference parcel in the anterior network. We selected the reference parcel to be on the opposite side of the cortical surface (in order to avoid influences from local noise correlations) and to be as far anterior as possible; for dorsal parcels on the lateral surface (overlapping OPA and cIPL), the reference parcel overlapped RSC on the medial surface; and for ventral parcels on the medial surface (overlapping PPA), the reference parcel overlapped cIPL on the lateral surface. In both cases, we observed rapid increases in connectivity as we moved posterior to anterior across the network boundaries (Fig. 2b,*c*). Along the dorsal boundary, we see significant increases in connectivity to the RSC parcel when moving from the first to the second parcel (left: *t*_(19)_ = 6.98, *p* < 0.001; right: *t*_(19)_ = 6.35, *p* < 0.001; two-tailed paired *t* test), from the second to the third parcel (left: *t*_(19)_ = 7.72, *p* < 0.001; right: *t*_(19)_ = 6.16, *p* < 0.001), and from the third to the fourth parcel (right: *t*_(19)_ = 2.44, *p* = 0.025). We observe a similar significant (though less dramatic) increase in connectivity to the cIPL parcel when moving from the first to the second PPA parcel (left: *t*_(19)_ = 4.21, *p* < 0.001; right: *t*_(19)_ = 2.68, *p* = 0.015) and from the second to the third PPA parcel (right: *t*_(19)_ = 3.03, *p* = 0.007).

### Connectivity with the hippocampus

Since the anterior scene network overlaps with default mode regions, while the posterior scene network does not, we predict that the anterior network should be more connected to the hippocampus (Buckner et al., 2008). To test this hypothesis, we measured the functional correlation at rest between mean hippocampal activity and the mean activity in each parcel within the posterior and anterior scene networks. As shown in Figure 3, there is a dramatic difference in hippocampal connectivity for parcels in the posterior network (overlapping with OPA and posterior PPA) compared with the anterior network (overlapping with RSC, cIPL, and anterior PPA). Moving posterior to anterior along the dorsal path, hippocampal connectivity first decreases slightly (first parcel to second parcel: left: *t*_(19)_ = −3.04, *p* = 0.007; right: *t*_(19)_ = 2.15 *p* < 0.04; two-tailed paired *t* test), then increases significantly when moving to the third parcel (left: *t*_(19)_ = 5.62, *p* < 0.001; right: *t*_(19)_ = 3.79, *p* = 0.001) and to the fourth parcel (left: *t*_(19)_ = 4.17, *p* < 0.001; right: *t*_(19)_ = 5.74, *p* < 0.001). Along the ventral path, hippocampal connectivity jumps from the first to the second parcel overlapping with PPA (left: *t*_(19)_ = 5.27, *p* < 0.001; right: *t*_(19)_ = 5.76, *p* < 0.001) and from the second to the third parcel (right: *t*_(19)_ = 5.80, *p* < 0.001).

We also investigated whether this effect was being driven by a subregion of the hippocampus, by correlating the mean time course in both scene networks with the time courses of each posterior-to-anterior coronal slice of the hippocampus. Our results show that the entire hippocampus is more strongly connected to the anterior scene–network than the posterior scene–network, but this difference is especially large in the anterior hippocampus. To confirm this pattern of results, we divided the hippocampus into posterior and anterior subregions at MNI coordinate *y* = −21 (Poppenk et al., 2013; Zeidman et al., 2015) and correlated their mean time courses with the two scene–network time courses. This analysis confirmed that the anterior network is more strongly connected to both posterior (*t*_(19)_ = 7.66, *p* < 0.001; two-tailed paired *t* test) and anterior (*t*_(19)_ = 6.58, *p* < 0.001) hippocampus than is the posterior scene network, and that this anterior–network connectivity is larger in anterior hippocampus (*t*_(19)_ = 3.29, *p* = 0.004); a repeated-measures ANOVA shows significant main effects of both hippocampal subregion (*F*_(1,19)_ = 11.32, *p* = 0.003) and scene network (*F*_(1,19)_ = 59.2, *p* < 0.001), and an interaction (*F*_(1,19)_ = 7.03, *p* = 0.016). Group-level connectivity values are reported in Table 1. Note that both the anterior and posterior scene networks are closer to posterior hippocampus, ruling out a distance-based explanation for this pattern of results.

**Table 1.** Anterior and posterior hippocampus connectivity to scene parcels.

	Dorsal										Ventral					
	1 (pSN)		2 (pSN)		3 (aSN)		4 (aSN)		5 (aSN)		A (pSN)		B (pSN)		C (aSN)	
	L	R	L	R	L	R	L	R	L	R	L	R	L	R	L	R
pHipp	−0.05	−0.06	−0.06	−0.06	0.02	−0.01	0.08	0.06	0.13	0.12	0	0.01	0.08	0.06	0.08	0.11
aHipp	−0.03	−0.03	−0.08	−0.07	0.06	0.02	0.13	0.11	0.22	0.20	0.03	0.07	0.11	0.09	0.12	0.15

For each scene–network parcel (as labeled in Fig. 3), the group-level connectivity (Fisher-transformed Pearson correlation) was calculated separately for posterior and anterior hippocampus. Parcels in the posterior scene network have lower hippocampal connectivity than those in the anterior scene network, especially for the anterior hippocampus. aHipp, anterior hippocampus; aSN, anterior scene network; L, left; pHipp, posterior hippocampus; pSN, posterior scene network; R, right.

### Comparison to meta-analyses and retinotopic atlas

**Figure 4.**
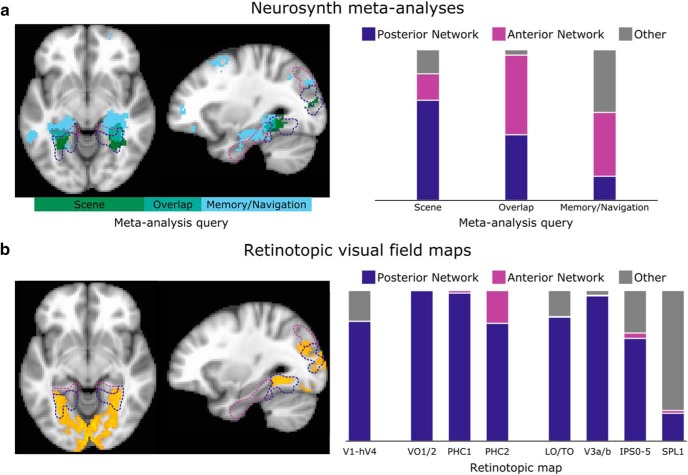
Overlap of posterior and anterior scene networks with previous work. ***a***, Two meta-analyses conducted using NeuroSynth identified overlapping but distinct reverse-inference maps corresponding to studies of visual scenes and to studies of higher-level memory and navigation tasks. These maps separate into our two scene networks, with visual scenes activating voxels in the posterior network and memory/navigation tasks activating voxels in the anterior network, as shown on example axial (*z* = −8) and sagittal (*x* = −30) slices. False discovery rate < 0.01; cluster size, 80 voxels (640 mm^3^). ***b***, Voxels having a >50% chance of belonging to a retinotopic map (orange) overlap with much of the posterior scene network, but end near the border of the anterior scene network. Breaking up the contributions of individual regions, we find that the probability mass of the topographic maps falls primarily within the posterior network, with only PHC2 showing a small overlap with the anterior network (probabilistically at the group level).

The connectivity results described thus far suggest a functional division for scene-related regions, with some belonging to a posterior network and others belonging to an anterior network. To assess the functional significance of these two networks, we ran two reverse-inference meta-analyses using the NeuroSynth tool (Yarkoni et al., 2011). This system automatically extracts activation coordinates from many fMRI studies (>10,000 at the time of writing); given a particular set of studies, it can identify voxels that are more likely to be activated in this set of studies relative to the full set of studies. These voxels are therefore preferentially active in the query set compared with general fMRI experiments. Based on the areas involved, we hypothesized that the posterior network processes the current visual properties of the scene, whereas the anterior network incorporates episodic memories and contextual aspects of the scene. Thus, in Figure 4a, we compare meta-analyses for the query “scene” (47 studies) with the query “episodic memory, navigation, past future” (125 studies). Along the parahippocampal gyrus, we find that the visual scene activations tend to be posterior to the memory activations, and that the transition point corresponds almost exactly to the division between our two networks. Dorsally, we also observe a separation between the reverse inference maps, with scene and memory activations falling into our two separate networks. Overall, voxels significant only in the scene meta-analysis were concentrated in the posterior network (66% in posterior network, 18% in anterior, 16% in other), while voxels significant only in the memory/navigation meta-analysis were spread more widely across the cortex, but were concentrated more in the anterior than the posterior network (16% posterior, 42% anterior, 42% other). Voxels significant in both the scene and memory/navigation meta-analyses tended to fall near the border between the two networks and divided approximately equally among them (44% posterior, 53% anterior, 4% other).

Another prediction of our framework is that voxels whose activity is tied to specific locations in the visual field (i.e., retinotopic) should, as clearly visual voxels, be present only in the posterior scene network. In Figure 4b, we compared our networks to a group-level probabilistic atlas of retinotopic visual field maps (Wang et al., 2014). The vast majority of the probability mass in this atlas is concentrated in the posterior network. In early visual cortex (V1, V2, V3, hV4), all nonfoveal portions of the visual field maps fall in the posterior network (80% posterior, 0% anterior, 20% other). Ventrally, the posterior network covers VO1/2 (100% posterior, 0% anterior, 0% other), PHC1 (98% posterior, 2% anterior, 0% other), and the peak of the probability distribution for PHC2, which also extends slightly across the anterior network border (78% posterior, 22% anterior, 0% other). Laterally and dorsally, the posterior network includes most of the LO1/2 and TO1/2 maps (82% posterior, 0% anterior, 17% other), V3a and V3b (96% posterior, 0% anterior, 3% other), and IPS0–IPS5 (68% posterior, 4% anterior, 28% other), with SPL1 being the only map falling substantially outside the networks that we consider (18% posterior, 2% anterior, 80% other).

## Discussion

**Figure 5.**
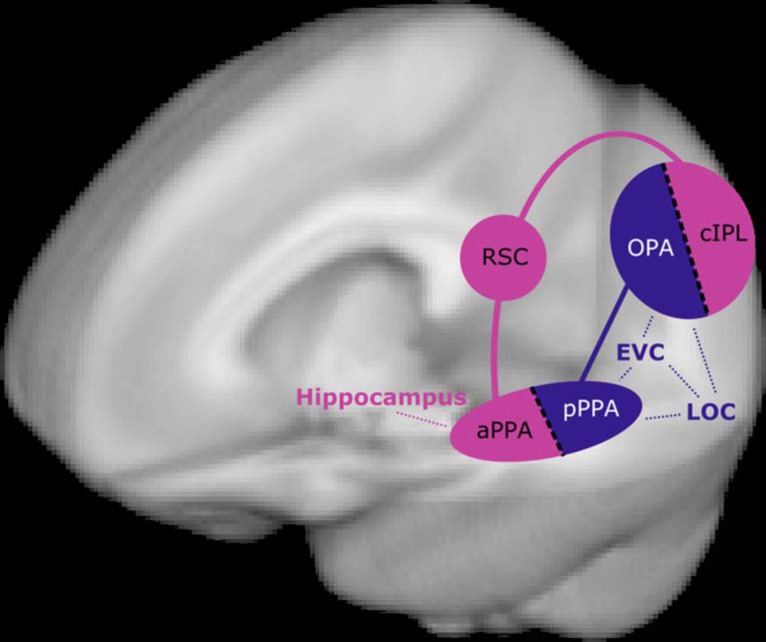
Two-network model of scene perception. Our results provide strong evidence for dividing scene-sensitive regions into two separate networks. We argue that OPA and posterior PPA (PHC1/2) process the current visual features of a scene [in concert with other visual areas, such early visual cortex (EVC), and LOC], while cIPL, RSC, and aPPA perform higher-level context and navigation tasks (drawing on long-term memory structures including the hippocampus).

By combining a variety of data sources, we have shown converging evidence for a functional division of scene-processing regions into two separate networks (summarized in Fig. 5). The posterior visual network covers retintopically organized regions, including OPA and pPPA, while an anterior memory-related network connects cIPL, RSC, and aPPA. This division emerges from a purely data-driven network clustering, suggesting that this is a core organizing principle of the visual system.

### Subdivisions of the PPA

The division of the PPA into multiple anterior–posterior subregions with differing connectivity properties replicates previous work (Baldassano et al., 2013) on an entirely different large-scale dataset, and shows that there is a strong connection between connectivity changes in PPA and the boundaries of retinotopic field maps. There is now a growing literature on anterior versus posterior PPA, including not only connectivity differences (Nasr et al., 2013; Silson et al., 2016a) but also the response to low-level (Nasr et al., 2014; Silson et al., 2015; Baldassano et al., 2016a,b; Watson et al., 2016) and high-level (Park et al., 2014; Aminoff and Tarr, 2015; Linsley and Macevoy, 2015; Marchette et al., 2015) scene features, as well as stimulation studies (Rafique et al., 2015). Our results place this division into a larger context, and demonstrate that the connectivity differences within PPA are not just an isolated property of this region but a general organizing principle for scene-processing regions.

### The visual network

The visual network shows a close correspondence with the full set of retinotopic maps identified in previous studies (Brewer and Barton, 2012; Huang and Sereno, 2013; Wang et al., 2014). Previous measurements in individual subjects have also shown strong overlap between OPA and retinotopic maps, especially V3b and LO2 (Nasr et al., 2011; Bettencourt and Xu, 2013; Silson et al., 2016a), and between pPPA and VO2, PHC1, and PHC2 (Arcaro et al., 2009). The only portion of cortex with known retinotopic maps that is not clustered in this network is the shared foveal representation of early visual areas, which segregates into its own cluster, which is consistent with other work showing a peripheral eccentricity bias in the scene network (Malach et al., 2002; Goesaert and Op de Beeck, 2010; Huang and Sereno, 2013; Baldassano et al., 2016a).

OPA and posterior PPA have been shown to be closely related to the visual content of a stimulus. Even low-level manipulations of spatial frequency (Rajimehr et al., 2011; Kauffmann et al., 2015; Watson et al., 2016) or rectilinearity (Nasr et al., 2014) can drive responses in these regions. Higher-level visual features also drive response patterns in these regions (Bryan et al., 2016), and they are hypothesized to be involved in extracting visual environmental features that can be used for navigation (Marchette et al., 2015; Julian et al., 2016; Kamps et al., 2016). However, neither OPA nor posterior PPA show reliable familiarity effects (Epstein et al., 2007b; see further discussion below).

The functional distinction between pPPA and OPA is currently unclear. Previous work has speculated about the purpose of the apparent ventral and dorsal “duplication” of regions sensitive to large landmarks, proposing that it may be related to different output goals (e.g., action planning in OPA, object recognition in pPPA; Konkle and Caramazza, 2013), or to different input connections (e.g., lower visual field processing in OPA, upper visual field processing in pPPA; Kravitz et al., 2013; Silson et al., 2015). OPA and pPPA may also use information from different visual eccentricities, with OPA processing less peripheral, relatively high-resolution environmental features and pPPA processing more peripheral, large-scale geometry, and context (Baldassano et al., 2016a).

### The memory and navigation network

The network of parahippocampal, retrosplenial, and posterior parietal regions that we identify has been emerged independently in many different fields of neuroimaging, outside of scene perception. Meta-analyses of internally directed tasks, such as theory of mind, autobiographical memory, and prospection, have identified this as a core, reoccurring network [Spreng et al., 2009; Kim, 2010; Yeo et al., 2015 (component C10 of )]. It comprises a subset of the broader default mode regions, but functional and anatomical evidence suggests that it is a distinct, coherent subnetwork (Andrews-Hanna et al., 2010, 2014; Yeo et al., 2011). The broad set of tasks that recruit this network have been summarized in various ways, such as “scene construction” (Hassabis and Maguire, 2007), “mnemonic scene construction” (Andrews-Hanna et al., 2010), “long-timescale integration” (Hasson et al., 2015), or “relational processing” (Eichenbaum and Cohen, 2014). A review of memory studies referred to this network as the posterior medial memory system, and proposed that it is involved in any task requiring “situation models” relating entities, actions, and outcomes (Ranganath and Ritchey, 2012).

The network has strong functional connections to the hippocampus, which has been implicated in a broad set of cognitive tasks involving “cognitive maps” for organizing declarative memories, spatial routes, and even social dimensions (Eichenbaum and Cohen, 2014; Schiller et al., 2015). During perception, the hippocampus binds together visual elements of an image (Olsen et al., 2012; Warren et al., 2012; Zeidman et al., 2015), which is especially important for scene stimuli (Lee et al., 2005a,b; Graham et al., 2006; Hodgetts et al., 2016) and then stores this representation into long-term memory (Ryan and Cohen, 2004). As we become familiar with an environment, the hippocampus builds a map of the spatial relationships between visual landmarks, which is critical for navigation (Morgan et al., 2011). Recalling or even imagining scenes also engages the hippocampus, especially anterior hippocampus, which may serve to integrate memory and spatial information (Zeidman and Maguire, 2016). Our results suggest that only the anterior scene regions interface directly with the hippocampus, potentially enabling the construction of hippocampal environmental representations, and retrieval of relevant memories and navigational information for a presented or imagined scene.

The specific functions of the individual components of this network have also been studied in a number of contexts. RSC appears to be most directly involved in orienting the viewer to the structure of the environment (both within and beyond the borders of the presented image) for the purpose of navigational planning; it encodes both absolute location and facing direction (Vass and Epstein, 2013; Epstein and Vass, 2014; Marchette et al., 2014), integrates across views presented in a panoramic sequence (Park and Chun, 2009), and shows strong familiarity effects (Epstein et al., 2007a,b). This is consistent with rodent neurophysiological studies, which have identified head direction cells in this region (Chen et al., 1994). RSC is not sensitive to low-level rectilinear features in nonscene images, such as objects or textures (Nasr et al., 2014), though it does show some preference for rectilinear features in images of 3D scenes (Nasr et al., 2014; Watson et al., 2016).

The specific properties of anterior PPA have been less well studied, since it was not recognized as a separate region within the PPA until recently. It has been shown to be driven more by high-level category information than by spatial frequency content (Watson et al., 2016), to represent real-world locations (even from perceptually distinct views; Marchette et al., 2015), to encode object co-occurrences (Aminoff and Tarr, 2015), and to represent real-world physical scene size (Park et al., 2014). Its representation of scene spaciousness draws on prior knowledge about the typical size of different scene categories, since it is affected by the presence of diagnostic objects (Linsley and Macevoy, 2015).

The cIPL (also referred to as posterior IPL, PGp, or the angular gyrus) has been proposed as a “cross-modal hub” (Andrews-Hanna et al., 2014) that connects visual information with other sensory modalities as well as knowledge of the past. It is more intimately associated with visual cortex than most lateral parietal regions, since it has strong anatomical connections to higher-level visual regions in humans and macaques (Caspers et al., 2011), and has a neurotransmitter receptor distribution similar to V3v and is distinct from the rest of the IPL (Caspers et al., 2013). It has been mostly ignored in the scene perception literature, primarily because it is not strongly responsive to standard scene localizers that show sequences of unfamiliar and unrelated scene images. For example, a study showing familiarity effects in cIPL described this location only as “near TOS” (Epstein et al., 2007b). The cIPL appears commonly, however, in studies involving personally familiar places, which are associated with a wealth of memory, context, and navigational information. It is involved in memory for visual scene images (Montaldi et al., 2006; Takashima et al., 2006; Elman et al., 2013; van Assche et al., 2016), learning navigational routes (Burgess et al., 2001; Bray et al., 2015), and even imagining past events or future events in familiar places (Hassabis et al., 2007; Szpunar et al., 2009). It can integrate information across space (Livne and Bar, 2016) and time (Lerner et al., 2011; Vilberg and Rugg, 2012), and has been shown in lesion studies to be critical for orientation and navigation (Kravitz et al., 2011). Our connectivity results and meta-analysis suggest that cIPL may play a prominent role in connecting visual scenes to the real-world location they depict.

### Contrasting the two networks

Although our work is the first to propose the visual versus context networks as a general framework for scene perception, several previous studies have shown differential effects within these two networks. Contrasting the functional connectivity patterns of RSC versus OPA or lateral occipital cortex (LOC; Nasr et al., 2013) or anterior versus posterior PPA (Baldassano et al., 2013) show a division between the two networks, consistent with our results. Contrasting scene-specific activity with general (image or word) memory retrieval showed an anterior versus posterior distinction in PPA and cIPL/OPA, with only more anterior regions (aPPA and cIPL, along with RSC) responding to content-independent retrieval tasks (Johnson and Rugg, 2007; Fairhall et al., 2014). Our two-network division is also consistent with the “dual intertwined rings” model, which argues for a high-level division of cortex into a sensory ring and an association ring, the second of which is distributed but connected into a continuous ring through fiber tracts (Mesmoudi et al., 2013).

### Open questions

The anterior/posterior pairing of aPPA/pPPA and cIPL/OPA raises the question of whether there is a similar anterior/posterior division in RSC. Evidence for a division has been mixed: wide-field retinotopic mapping using natural scenes shows a partial retinotopic organization in RSC (Huang and Sereno, 2013); the response of RSC to visual rectilinear features appears to be limited to the posterior portion (Nasr et al., 2014); but a study of retinotopic coding in scene-selective regions failed to find any consistent topographic organization to RSC responses (Ward et al., 2010), and previous analyses of the functional properties of anterior versus posterior RSC have not found any significant differences (Park et al., 2014). A very recent study (Silson et al., 2016b) that carefully compared scene selectivity, functional connectivity, and retinotopic mapping has proposed that there are in fact two separable subregions in medial parietal cortex. The more anterior region is strongly connected to anterior PPA and is less retinotopic, likely corresponding to the parcel overlapping RSC on which we focus in this work. The more posterior region, which falls in the parieto-occipital sulcus, is more strongly driven by visual scenes, has a clear contralateral field bias, and is connected more evenly to the subregions of PPA (though still more to anterior than posterior PPA). Future work may confirm that this region should also be included as a part of the visual scene network, yielding a third interface between the two networks.

Another interesting question is how spatial reference frames differ between and within the two networks. Given its retinotopic fieldmaps, the visual network presumably represents scene information relative to the current eye position; previous work has argued that this reference frame is truly retina centered and not egocentric (Ward et al., 2010; Golomb and Kanwisher, 2012). The context network, however, likely transforms information between multiple reference frames. Models of spatial memory suggest that medial temporal lobe (possibly including aPPA) uses an allocentric representation, while the posterior parietal lobe (possibly including cIPL) is based on an egocentric reference frame, and that the two are connected via a transformation circuit in RSC that combines allocentric location and head direction (Byrne et al., 2007; Vann et al., 2009). There is some recent evidence for this model in human neuroimaging: posterior parietal cortex codes the direction of attention in an egocentric reference frame (even for positions outside the field of view; Schindler and Bartels, 2013), and RSC contains both position and head direction information (anchored to the local environment; Marchette et al., 2014; Shine et al., 2016). This raises the possibility that another critical role of cIPL could be to transform retinotopic visual information into a stable egocentric scene over the course of multiple eye movements. The properties of aPPA, however, are much less clear; it seems unlikely that it would use an entirely different coordinate system than neighboring PHC1/2, and some aspects of the scene encoded in aPPA, such as object co-occurrence (Aminoff and Tarr, 2015), do not seem tied to any particular coordinate system.

Finally, we note that a hard division into two networks is only a first-order description of the structure and function of scene regions. A number of these regions (e.g., PHC2) fall on a continuum from visual to contextual, and recent theories of information processing argue that almost all cortical regions accumulate information at varying timescales (Hasson et al., 2015). Task demands will also shift the functions of these regions (e.g., during top-down imagery; Dentico et al., 2014) and can lead to the dynamic reconfiguration of networks (Bray et al., 2015). Our proposed framework is intended to capture the primary functional dimension that distinguishes between scene-sensitive regions during natural perception, and to offer a starting point for future work on the organization of the human scene-processing system.

### Conclusion

Based on data-driven connectivity analyses and analysis of previous literature, we have proposed a unifying framework for understanding the neural systems involved in processing both visual and nonvisual properties of natural scenes. This new two-network classification system makes explicit the relationships between known scene-sensitive regions, re-emphasizes the importance of the functional subdivision within the PPA, and incorporates posterior parietal cortex as a primary component of the scene-understanding system. Our proposal that much of the scene-processing network relates more to contextual and navigational information than to specific visual features suggests that experiments with unfamiliar natural scene images will give only a partial picture of the neural processes evoked in real-world places. Experiencing our visual environment requires a dynamic cooperation between distinct cortical systems to extract information from the current view of a scene, and then to integrate it with our understanding of the world and determine our place in it.

*Note added in Proof* - Minor revisions were made to the version that was published on-line October 10, 2016, as an Early Release, including adjustments to the labeling of Figures 2 and 3, and small wording changes in the Abstract and Materials and Methods.

## Synthesis

The decision was a result of the Reviewing Editor Howard Eichenbaum and the peer reviewers coming together and discussing their recommendations until a consensus was reached. A fact-based synthesis statement explaining their decision and outlining what is needed to prepare a revision is listed below. The following reviewers agreed to reveal their identity: Peter Zeidman, Sean MacEvoy

Both reviewers judged the paper to make a valuable contribution to this literature. However, one of the reviewers had several recommendations for clarification and explanation that will improve the paper substantially. These recommendations should be give serious consideration in preparing the final manuscript.

Reviewer 1:

I enjoyed reviewing this paper. I feel the manuscript needs some further work - particularly reining back certain claims that cannot be made with the data available, as well as adding detail to the hippocampus connectivity results. I hope these suggestions will prove useful to the authors in revising the paper.

I would first like to make a general point, before discussing the specifics of the paper in more detail. The authors identified two networks, which they emphasise throughout the manuscript are "distinct" or "separate". While I think the division of the network into clusters is useful, their claiming that these networks are discrete is made too strongly. The data analysis was based on parcellation / clustering, which could only produce evidence of distinct regions / networks. We know the brain has hierarchies of processing, which are particularly well studied in the visual stream. Connectivity is characterised by both gradients and sharp distinctions (e.g. see Strange et al., NRN, 2014), and indeed the authors' own results speak to this. A linear trend could easily be fitted to the data graphed in Figure 2 or Figure 3b-c, suggesting the network divisions identified by the authors are not simply discrete. I suggest that the discussion needs to state more clearly that the discrete division between networks, which is a useful model imposed to help our understanding, exists in the context of cortical hierarchies and gradients of connectivity.

Methods p. 6-7

The authors performed their parcellation on data from 468 HCP subjects, but then did the remainder of their analyses on an arbitrary 20 subjects. I don't understand the reason for only using a small subset of subjects for the analyses. The methods section states that "data [from 20 subjects] was used to statistically measure the robustness of connectivity differences observed in the group-level data". Robustness generally refers to violations of the assumptions of a statistical model - what assumption were the authors referring to here? I am unclear how looking only at the first 20 subjects could help in this respect.

Methods - "Scene localizers" p.8

The degree of overlap between scene regions (e.g. PPA) and the network parcellation depends on the size of the masks defined by the authors in their localiser study. The mask sizes appear to have been defined arbitrarily - e.g. the top 300 voxels for PPA at the single subject level. This was then taken to the group level, after which I'm not clear what criteria was used to define the edges of the masks. The sentence "the cluster denoting the highest overlap between subjects was then manually annotated" is unclear to me. The details of how the masks were defined and the rationale behind it needs to be better explained in the manuscript. In particular, I wonder why the authors didn't follow the conventional approach of performing a group level mass-univariate GLM analysis on the scene localiser data and including voxels in the masks which exceeded some statistical threshold.

Methods - "Parcel-to-Parcel... connectivity" p. 9

Just to make this section clearer - could the authors be very specific about what they meant by "correlating the mean eigenmaps" on lines 151-152.

Results - "Clustering parcels into networks" p . 12

It would be helpful if the authors could describe in anatomical terms the location of the anterior and posterior PPA they have found - i.e. the gyri and sulci covered by the regions.

The authors frame the "connectivity shifts across the network border analysis" as selecting two arbitrary regions from their anterior network (RSC and cIPL) for analysis. Why were these particular regions selected? Were they in fact particularly interesting regions, or were they arbitrarily selected as stated? If the former, this should be explained - e.g. there may be very good reason why OPA/cIPL coupling with RSC is interesting for understanding scene processing. If the latter, it would make more sense to detail the connectivity of the three seeds in PPA (Fig 2b) with separate graphs for several of the scene regions, rather than picking two at random.

Figure 1 p. 14

I initially found this confusing and a couple of modifications to the legend would make things clearer. I suggest after the first sentence, adding words to the effect of "Parcels illustrated with the same color were assigned to the same cluster in a second hierarchical clustering analysis..." And in the final sentence, I suggest saying that OPA and posterior PPA were identified using a scene localiser in a separate set of subjects.

Results - "Connectivity with the hippocampus" p. 16

I feel this section of the paper is a key novel contribution which will be useful to many researchers who focus on the human hippocampus. However, I think the reporting of the results needs to be improved to bring out some of the most interesting aspects of the data. First, the stated motivation for this analysis and conclusions that can be drawn from it need to be revised. The authors motivated this analysis as follows: "we predicted that the anterior network should be more related to memory and navigation tasks that engage the hippocampus. To test this hypothesis...". This study only used resting state data and a passive scene viewing task - the authors could not have tested any hypotheses specifically regarding memory or navigation. This claim should be removed from the results section, as well as in the interim conclusion on line 274, which reads "This elevated hippocampal connectivity in the anterior network is consistent with our hypothesis that the anterior network is more closely related to navigation and memory". The hippocampus connectivity analysis is a very nice contribution without needing to speak to specific cognitive functions. Speculating on specific functions associated with the brain regions involved is welcome, but this should be limited to the discussion, not the results section.

Please could the authors also clarify in the manuscript which dataset is being used for the hippocampus analysis? I assume the first 20 subjects from the HCP?

The authors shows a very interesting gradient of connectivity that peaks in anterior hippocampus. However, there's no way to know from the results which specific cortical regions contributed to this result. I think a more detailed breakdown of the results would make the paper more useful. I suggest the following revised structure, at the author's discretion. Start with the graph currently in Figure 3d, demonstrating a clear difference between anterior and posterior hippocampus. Then perform the analyses currently appearing in Figure 3a-c separately for anterior and posterior hippocampus. (At present the authors collapse over the long axis of the hippocampus, which doesn't make sense given that they then go on to show a clear distinction between anterior and posterior hippocampus connectivity.) It may also add anatomical detail to show the coronal slices with correlations overlaid (potentially in supplementary material).

Readers may wish to use the authors' results from this section, and from the previous section, to quantitatively guide future analyses. Could the authors provide a full correlation matrix between parcels and anterior hippocampus, and between parcels and posterior hippocampus? This could be in tables, in supplementary material or via a data repository like openfmri.org? This would be very useful for the wider community.

"Comparison to Meta-analyses and retinotopic atlas" - p. 19

I am not convinced by the NeuroSynth analysis and I think it lets the rest of the paper down. The authors compared the results for the query "scene" against "episodic memory OR navigation OR past future" (perhaps a missing 'OR' in there?). The interpretation of the results in the next sentence reads "Along the parahippocampal gyrus, we find that the visual scene activations to be posterior...". The authors did not query NeuroSynth for visual scenes and so cannot conclude this. More generally, the results of the "scene" query gives studies looking at visual scene perception, imagination, future thinking, memory, navigation - greatly overlapping with the second query they performed. The results are therefore very hard to interpret and I don't think contribute to the paper. I suggest that the NeuroSynth analysis is thoroughly revised or removed from the paper entirely. By contrast, I think the results of the retinotopic analysis are very impressive and should be emphasised.

Figure 5 - p. 22

Perhaps the hippocampus should be plugged into the networks in this figure?

Discussion - p. 26

Following from my comments above, I feel the authors must remove the claim on line 458 "Our results argue that only the anterior scene regions are directly involved in building hippocampal representations of the environment, and in retrieving relevant memories and navigational information for a presented or imagined scene". This study results provide no evidence for these claims.

Reviewer #2:

This paper is a superb contribution to the literature, marking an important advance in our conceptualization of scene processing regions with its clear, comprehensive, and compelling argument that these regions are best understood as elements of two complementary networks. I believe that the framework laid out by this paper will become an important touchstone for research into scene processing in the future.

I have only a few comments:

1.When the authors state that distinction between the anterior and posterior networks is best understood as between contextual and visual processing, are they asking us to conceive of scene representations in the anterior network to be uniformly amodal?

2.Given the rapid shift in connectivity profiles between anterior and posterior aspects of PPA, one is left to wonder "Why are these two scene selective regions attached to each other?" Is this evolutionary baggage, or does it reflect a gradient critical to information processing (i.e., this is the interface between anterior and posterior networks). With that in mind, is it possible to express the connectivity of the two subregions of PPA to each other relative to their connections to other areas that the authors' analysis associates them with? Is this mutual connectivity stronger than between other areas on opposite sides of the anterior/posterior scene network divide?

Line 99: The use of the word "demeaning" in this way is beneath contempt! Is someone defiled when their filing cabinet is emptied?

I noticed a number places where the word "on" was used when "upon" might have been a better choice. I defer to the authors' judgment however.
